# Improved Tricyclic Inhibitors of Trypanothione Reductase by Screening and Chemical Synthesis

**DOI:** 10.1002/cmdc.200900097

**Published:** 2009-08-03

**Authors:** John L Richardson, Isabelle R E Nett, Deuan C Jones, Mohamed H Abdille, Ian H Gilbert, Alan H Fairlamb

**Affiliations:** a Dr. J. L. Richardson, Dr. I. R. E. Nett, Dr. D. C. Jones, M. H. Abdille, Prof. I. H. Gilbert, Prof. A. H. Fairlamb Division of Biological Chemistry & Drug Discovery, College of Life Sciences, University of DundeeDundee DD1 5EH, Scotland (UK), Fax: (+44) 1382-385-542

**Keywords:** drug discovery, inhibitors, oxidoreductases, *trypanosoma brucei*, trypanothione reductase

## Abstract

*Trypanothione reductase (TryR) is a key validated enzyme in the trypanothione-based redox metabolism of pathogenic trypanosomes and leishmania parasites. This system is absent in humans, being replaced with glutathione and glutathione reductase, and as such offers a target for selective inhibition. As part of a program to discover antiparasitic drugs, the LOPAC1280 library of 1266 compounds was screened against TryR and the top hits evaluated against glutathione reductase and* T. brucei *parasites. The top hits included a number of known tricyclic neuroleptic drugs along with other new scaffolds for TryR. Three novel druglike hits were identified and SAR studies on one of these using information from the tricyclic neuroleptic agents led to the discovery of a competitive inhibitor (*K*_i_=330 nm) with an improved potency against* T. brucei *(EC_50_=775 nm).*

## Introduction

Parasitic diseases are a major obstacle to human health and economic development in many parts of the world, and cause high rates of mortality and morbidity. In particular protozoa of the order Kinetoplastida are a significant problem. These parasites give rise to Chagas’ disease (caused by *Trypanosoma cruz*i), sleeping sickness (*Trypanosoma brucei spp*.), and leishmaniasis (*Leishmania spp.)*.^1^ These infections afflict between 28 and 30 million people, cause in excess of 120,000 deaths a year, and contribute to 4.4 million years of disability-adjusted life.^1^ At present therapies against these diseases are poor with treatment failure being common due to widespread resistance and severe side effects.^1^ Thus there is a need for the development of new, efficient and safe drugs for the treatment of these diseases.

**Scheme 1:**
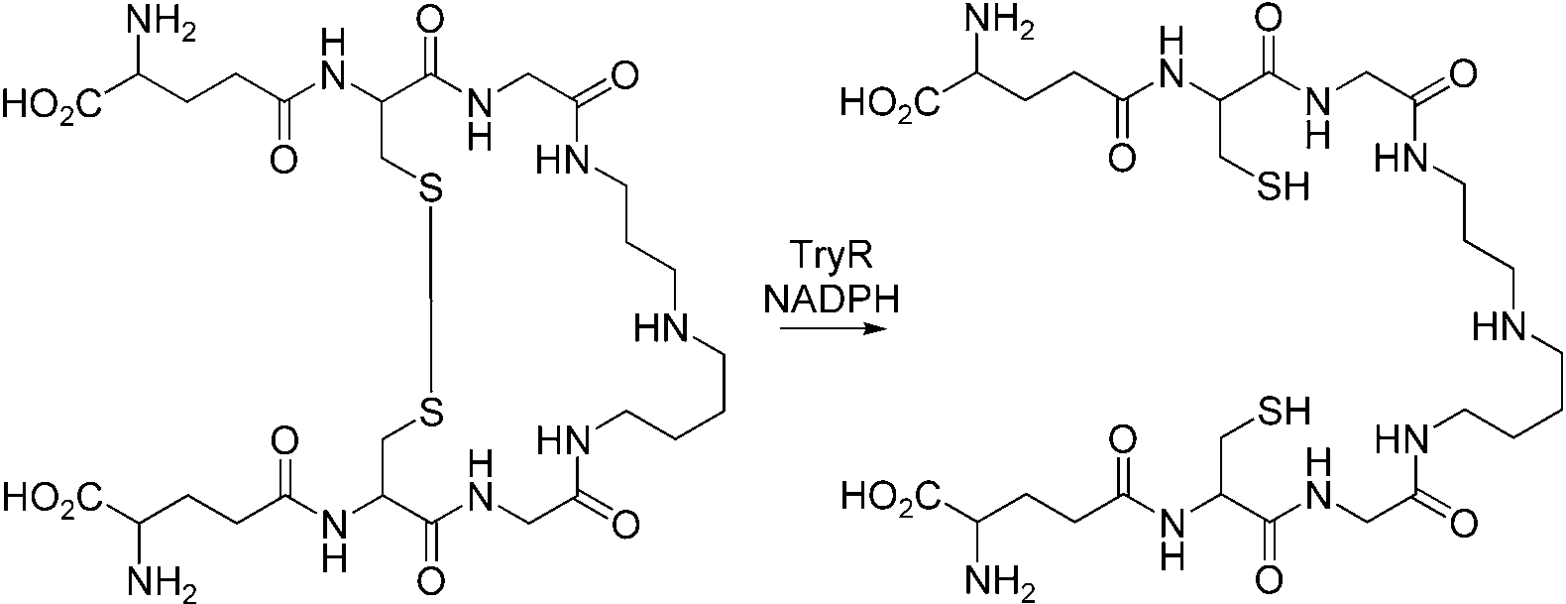
Reaction carried out by trypanothione reductase (TryR).

A promising target for the design of new drugs involves thiol metabolism in these protozoa, where redox balance is maintained uniquely by the dithiol trypanothione (T[SH]_2_ or *N*^1^,*N*^8^-bis[glutathionyl]spermidine).^2^ Trypanothione counteracts environmental oxidative stress through a variety of enzymatic and nonenzymatic reactions, and has been implicated in acquired resistance to chemotherapeutic agents.^3^, ^4^ The majority of these protective reactions oxidize T[SH]_2_ to trypanothione disulfide (T[S]_2_), which is recycled back to T[SH]_2_ by trypanothione reductase (TryR; EC 1.8.1.12), an NADPH-dependent disulfide oxidoreductase (Scheme 1). TryR is thought to be the central enzyme in the redox metabolism of these protozoans, being the sole route of reducing equivalents from the NADP^+^/NADPH couple to thiol-containing species.^5^ The importance of trypanothione metabolism is reflected in the fact that a number of currently used antiprotozoan compounds work, at least in part, by affecting trypanothione metabolism; the melamino-arsenicals bind to trypanothione and possibly also TryR;^6^ difluoromethylornithine (DFMO) inhibits the biosynthesis of spermidine, a constituent of trypanothione;^7^ and antimonials form complexes with trypanothione and inhibit TryR.^8^

Genetic validation (RNAi and knockout studies) has indicated the importance of TryR to parasite survival.^9^–^11^ In contrast, in the mammalian host, trypanothione metabolism is absent and glutathione is the primary low-molecular-mass thiol, with the enzyme glutathione reductase (GR) performing the role of reducing glutathione to glutathione disulfide. Despite trypanosomatid TryR and human GR both showing ≈40 % sequence identity,^12^ each enzyme is highly specific for its respective disulfide substrate, where charge is a major discriminating factor.^13^, ^14^ The significant structural differences between TryR and GR suggests that it should be possible to design selective inhibitors of TryR. Indeed, there are many cases of selective inhibitors of TryR reported in the literature, adding further weight to TryR as a potential drug target.

**Figure 1:**
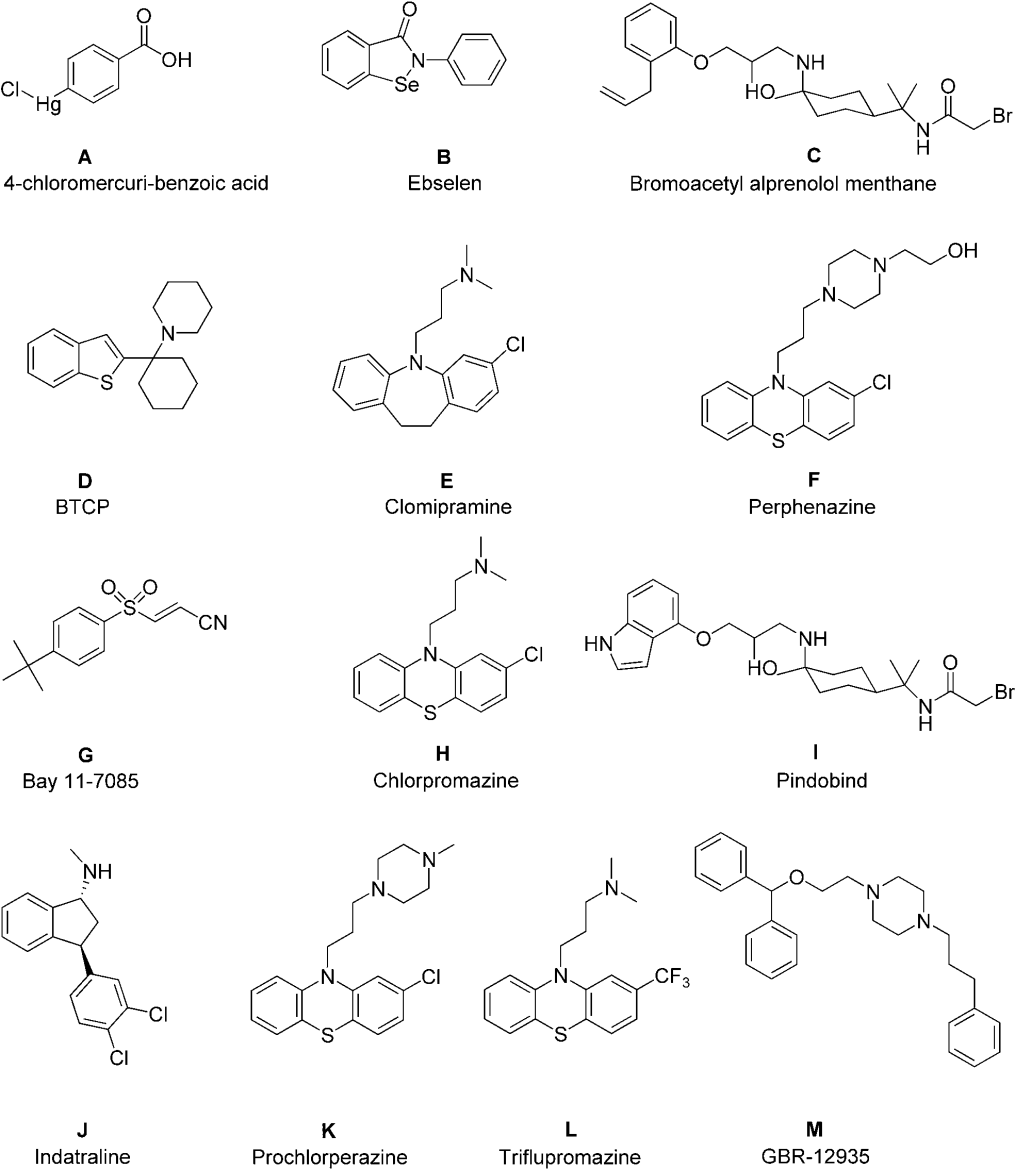
Structures and trivial names of screening hits (see Table 1).

In recent years several different classes of compounds have been reported as TryR inhibitors.^15^–^52^ However, with the exception of the antimonials,^8^ none of these compounds are currently used clinically against trypanosomiases or leishmaniases. To address the need for new compounds and new compound classes, we initiated a screening of 1266 pharmacologically active compounds from the Sigma–Aldrich LOPAC1280 library. These compounds were screened against TryR, and the top hits counter-screened against GR and live *T. bruce*i parasites, yielding the IC_50_ values, selectivity for TryR over GR and antiparasitic activity. We also investigated the druglikeness of the molecules.^53^ Based on these data we synthesised a number of analogues and fragments of one of the most promising hits: 1-(2-(benzhydryloxy)ethyl)-4-(3-phenylpropyl)piperazine (**M**) based on its similarity to the known tricyclic (neuroleptic) inhibitor of TryR, prochlorperazine (**K**) (Figure 1). Kinetic testing of these compounds revealed a fivefold improvement over **M** for one of them. Incorporating these improvements to prochlorperazine itself produced a competitive inhibitor with a tenfold improvement in IC_50_ value, with a *K*_i_ value in the submicromolar region. Subsequent testing on live *T. bruce*i parasites showed most of the compounds to be more active against the parasites than against the purified enzyme. The compound with the highest EC_50_ value was compound **9**.

## Results and Discussion

### Screening

The LOPAC1280 library, comprising 1266 compounds, was initially screened in duplicate at 100 μm yielding 170 active compounds with inhibition >50 % for TryR. Hits were re-tested at 10 μm and the 35 most potent compounds (inhibition>77 %) were assayed to determine IC_50_ values against TryR. The 22 most potent molecules also had their IC_50_ values determined against human GR and were assayed for trypanocidal activity in vitro.

**Figure 2:**
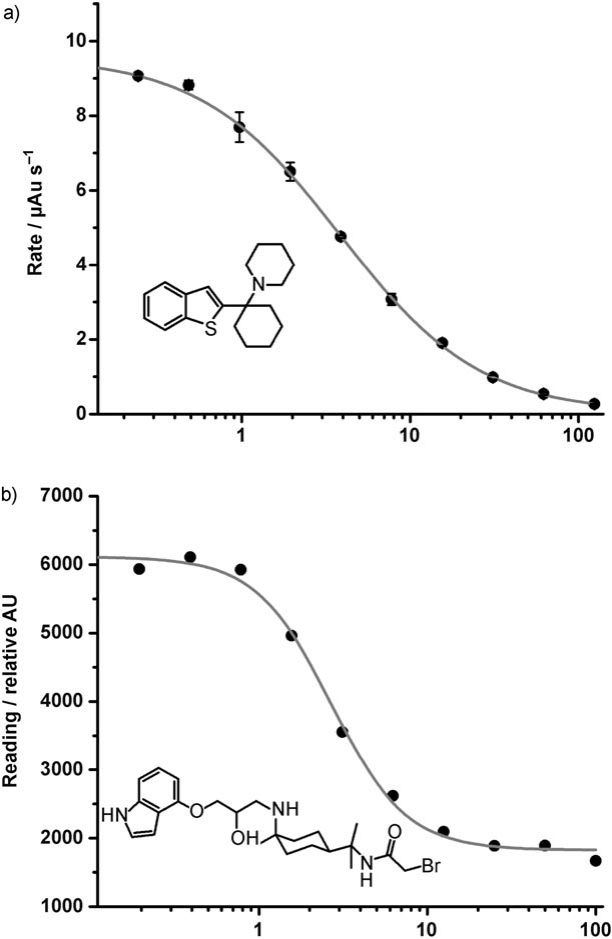
Specimen data showing a) IC_50_ determination for compound **D** against TryR (IC_50_=3.69±0.06 μ m) and b) EC_50_ determination of compound **I** against *T. brucei* parasites (EC_50_=2.68±0.15 nm). Experimental details are described in the Experimental Section. The curves show the average value of three independent measurements (•) and the best fit to a nonlinear four-parameter equation (—).

The most potent TryR inhibitor (aurin tricarboxylic acid; 3-(bis(3-carboxy-4-hydroxyphenyl)methylene)-6-oxocyclohexa-1,4-dienecarboxylic acid) had an IC_50_ value of 176 nm, but was not selective for TryR over GR and showed the weakest trypanocidal activity in the series. Results for the top 13 inhibitors of TryR, which also showed inhibition of *T. brucei* with EC_50_ values <15 μm, are summarised in Table 1 ranked in order of inhibition of TryR (see Figure 2 for typical IC_50_ and EC_50_ determinations). Of these, four compounds (**A**–**D**) had IC_50_ values less than clomipramine (**E**; IC_50_=3.8 μm), a known tricyclic (neuroleptic) competitive inhibitor of TryR, which happened to be present in the LOPAC1280 library.^15^, ^30^ The IC_50_ value for clomipramine **E** agrees well with the IC_50_ determined using our commercial standard (2.7 μm) demonstrating the robustness of the assay. When tested against the parasite, nine compounds had EC_50_ values less than clomipramine. The most potent trypanocidal compound **A** had an EC_50_ value of 1.5 nm, which is lower than the standard drug pentamidine (EC_50_=18 nm).^54^ However, it was disappointing to discover that compound **A** is an organo-mercurial, a nonspecific thiol-alkylating agent and therefore totally unsuitable as a lead. Indeed, analysis revealed that many of the best hits by IC_50_ and EC_50_ value were not druglike^53^ and the most druglike hits were from the already known tricyclic (neuroleptic) class of inhibitors.^15^, ^30^ Of the thirteen most active compounds in Table 1, nine have EC_50_ values against the parasite lower than their IC_50_ values against the target enzyme (i.e. ratio<1), which suggests that these compounds may have additional off-target effects (e.g. **A**, **B**, **C**, **G** and **I** have thiol-reactive groups and show the lowest selectivity index between TryR and GR), or may be selectively concentrated/metabolically activated in the parasite, or a combination of these.

**Table 1:** Top 13 results from EC_50_ and IC_50_ value determinations from a LOPAC1280 screen ranked in order of IC_50_ potency against TryR.

Compd	*T. brucei* EC_50_ [μm]	TryR IC_50_ [μm]	GR IC_50_ [μm]	*Tb* EC_50_/ TryR IC_50_	SI^[a]^	Drug Score^[b]^
**A**	0.00154	0.251	0.00501	0.00614	0.0200	–
**B**	2.97	1.36	21.5	2.18	15.8	–
**C**	0.139	2.21	3.37	0.0629	1.52	0.07
**D**	13.6	3.69	>100	4.39	>32.3	1.35
**E**	5.04	3.80^[c]^	>100	1.33	>26.3	1.29
**F**	7.67	4.88	>100	1.57	>20.5	1.79
**G**	1.52	4.94	12.5	0.308	2.53	−1.52
**H**	4.21	6.32	>100	0.666	>15.8	1.42
**I**	2.89	6.89	4.56	0.419	0.662	−0.13
**J**	1.97	7.26	>100	0.271	>13.8	0.65
**K**	4.48	7.46	>100	0.601	>13.4	1.83
**L**	1.73	7.53^[d]^	>100	0.230	>13.3	1.58
**M**	9.3	10.9	>100	0.853	>9.17	0.57

[a] Selectivity index: ratio of GR IC_50_ over TryR IC_50_. [b] Druglikeness scores calculated using the High Speed Molecular Properties calculator from Molsoft.^53^ Most known drugs have a score > 0 and the optimum score value is 1.0. [c] *K*_i_=6.5±0.6 Dixon plot 20 mm HEPES pH 7.25, 0.15 mm KCl.^15^ [d] *K*_i_=21.9±1.7 Dixon plot 20 mm HEPES pH 7.25, 0.15 mm KCl.^15^

Most compounds show specificity towards TryR with only two displaying preferential inhibition of GR (ratio GR IC_50_/TryR IC_50_ <1) and two displaying poor selectivity (ratio GR IC_50_/TryR IC_50_>1 and <3). Selectivity (SI) towards TryR marked as > in Table 1 indicates that these compounds showed less than 20 % inhibition of human GR at a concentration of 100 μm so that an accurate IC_50_ could not be obtained.

Interestingly, of the nine compounds in Table 1 that are greater than ninefold selective for the parasite enzyme, five belong to the tricyclic (neuroleptic) class of compounds.^3^ This is significant given that the target product profile for African sleeping sickness is for a compound that is active against late stage CNS infections, which could replace melarsoprol, an arsenical that causes fatal encephalopathy in about 5 % of patients.^1^ However, the archetypical drug of this class, clomipramine, has already been shown to be of marginal value as a trypanocidal drug lead.^33^

Another key filter for progression of compounds is druglikeness. This is usually investigated by calculating the physicochemical properties of a compound to ensure that they are appropriate to permit the inhibitor to reach the molecular target in a whole organism. For orally bioavailable compounds, Lipinski’s rule of five is used to assess this.^55^ The concept of druglikeness can be extended further to ensure that there are no chemically or metabolically reactive functionalities, although this often requires experimental determination. For HAT, the desired compound must have at least some degree of blood–brain barrier permeability in order to treat late-stage infections, which imposes additional constraints (ideally a lower molecular weight and polar surface area). As an approach to measure druglikeness, compounds were assessed using the molsoft drug-scoring system (http://www.molsoft.com). Of the top three hits as ranked by EC_50_ value against *T. brucei*, the druglikeness of **A**, **C** and **G** is poor, partly because of the chemical reactivity of these molecules. Likewise, ebselen (**B**) was not deemed suitable for further development, partly due to the fact it contained selenium, but also due to its reported reaction with thiol groups and NADPH in the presence of a thiol group containing enzyme,^56^, ^57^ suggesting that it might be a promiscuous inhibitor.

In summary, a number of criteria were applied to select hits from the initial screen: potency and selectivity against both enzyme and parasite; druglikeness, particularly the probability of blood–brain barrier permeability; and chemical tractability. This gave three compounds that we decided to investigate further: GBR-12935 (**M**), BTCP (**D**), and indatraline (**J**). (Substantial work has also been published around the tricyclic compounds as potential antitrypanosomals.^14^, ^15^, ^23^, ^29^, ^30^, ^39^) Herein, we describe the further study of compound **M** and derivatives as potential antiparasitic agents. Further data based on BTCP (**D**), and indatraline (**J**) will be described in due course.

### Chemistry

A limited chemistry programme was carried out around compound **M** (compounds **3**–**9**), in particular to see if there was any cross-over with this scaffold and that of clomipramine (**E**) and the phenothiazines (**F**, **H**, **K**, **L**). Thus, the diphenylmethane moiety was cyclised to the corresponding dibenzosuberol and phenothiazine scaffolds (**6**–**8** and **9**, respectively). Also the effect of the phenylpropyl substituent was investigated (cf. **6** and **7**).

**Scheme 2:**
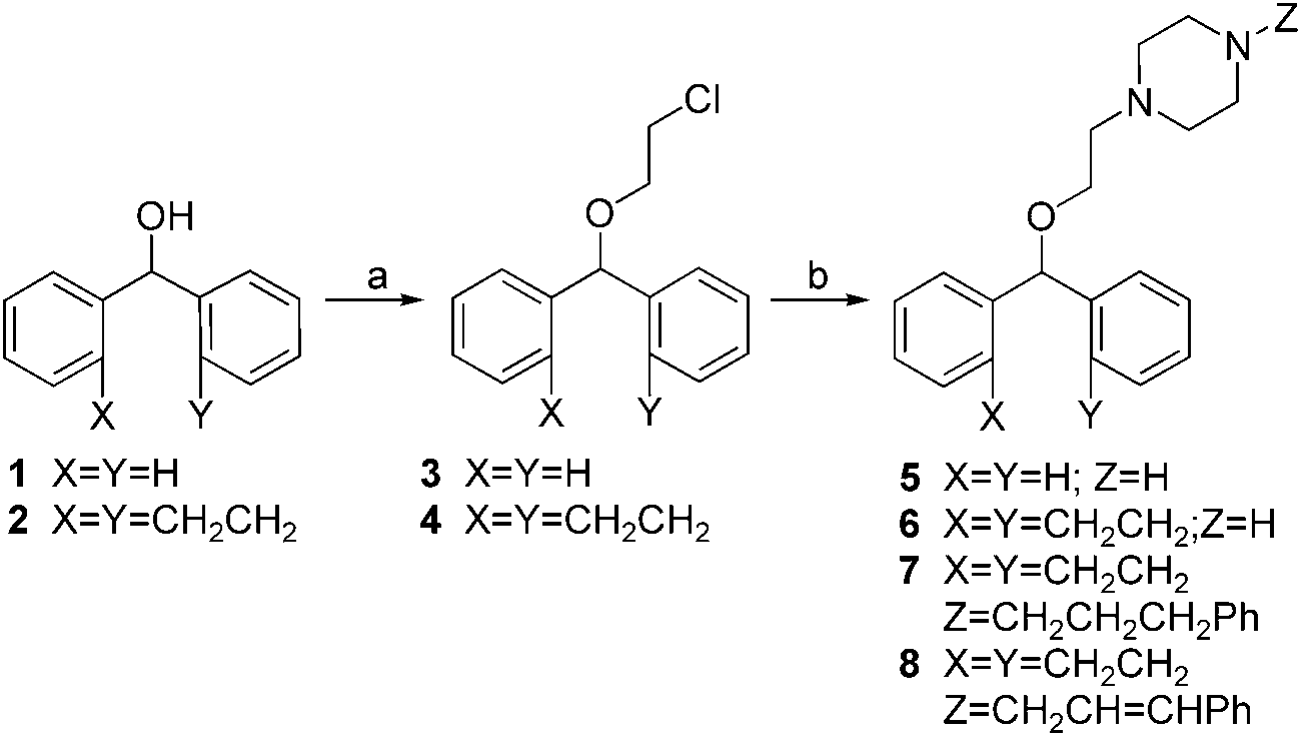
*Reagents and conditions*: a) 2-chloroethanol, *p*TSA, MePh, Δ; b) substituted piperazine, K_2_CO_3_, MePh, Δ.

The compounds **6**, **7** and **8** (Scheme 2) were prepared by nucleophilic substitution reactions between the intermediate 5-(2-chloroethoxy-)-10,11-dihydro-5*H*-dibenzo[*a,d*]cycloheptene (**4**) and the appropriate substituted piperazines in yields of between 33 and 41 %.^58^ Compound **5** was prepared by nucleophilic substitution between the intermediate (2-chloroethoxy)diphenylmethane **3** and anhydrous piperazine (Scheme 2) in a yield of 53 %.^59^ Intermediate compound **4** was formed by reaction between dibenzosuberol and 2-chloroethanol in a yield of 70 %. Similarly, compound **3** was formed by reaction of benzhydrol and 2-chloroethanol in a yield of 82 %.^58^ The substituted piperazine, 1-(3-phenylpropyl)piperazine, was formed by a nucleophilic substitution reaction between anhydrous piperazine and 1-bromo-3-phenylpropane in a yield of 23 %.

**Scheme 3:**
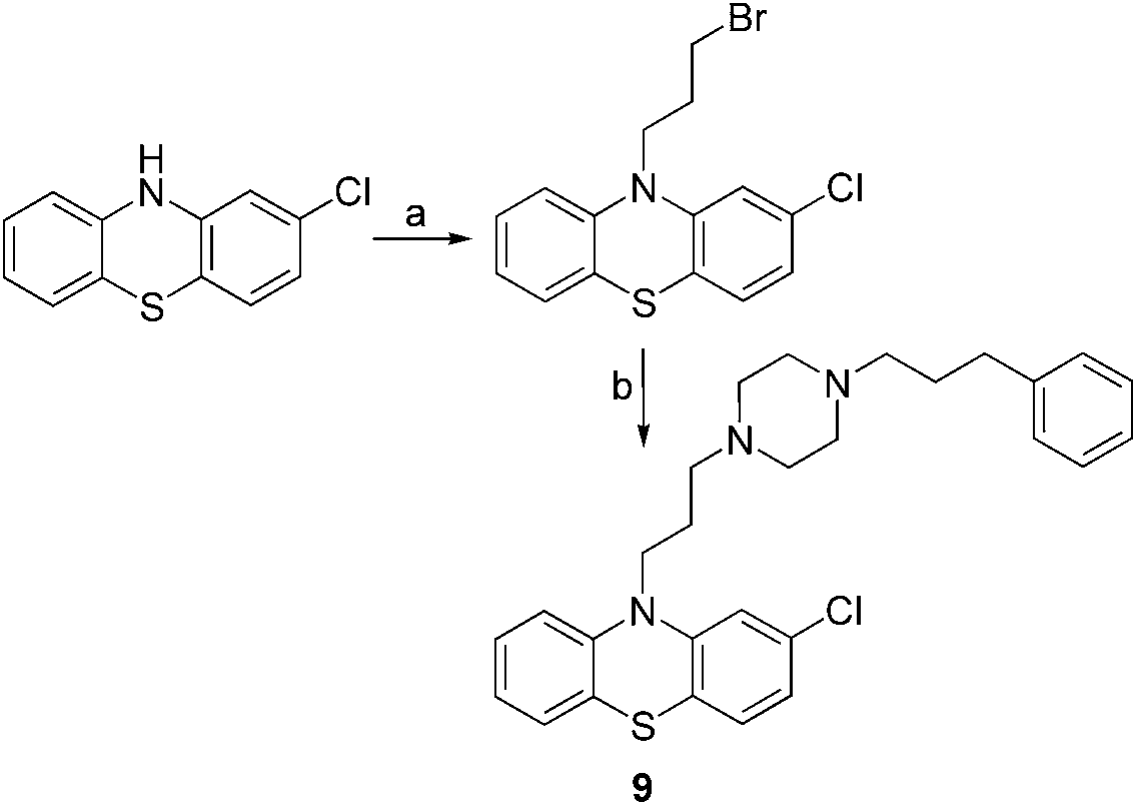
Synthesis of 2-chloro-10-(3-(4-(3-phenylpropyl)piperazin-1-yl)propyl)-10*H*-phenothiazine (**9**). *Reagents and conditions*: a) BrCH_2_CH_2_CH_2_Br, KOH, DMF; b) 1-(3-phenylpropyl)piperazine, K_2_CO_3_, MePh, Δ.

The synthesis of 2-chloro-10-(3-(4-(3-phenylpropyl)piperazin-1-yl)propyl)-10*H*-phenothiazine (**9**, Scheme 3) was achieved in two steps by nucleophilic substitution between 2-chloro-10*H*-phenothiazine and 1,3-dibromopropane to give 10-(3-bromopropyl)-2-chloro-10*H*-phenothiazine.^60^ Further reaction, without prior purification, with 1-(3-phenylpropyl)piperazine afforded **9** in an overall yield of 5 %.

### Enzymatic assays

Cyclisation of the diphenyl derivative **M** to the tricyclic scaffolds based on clomipramine (**E**) or chlorpromazine (**H**) gave an improvement of activity against TryR (**7**, IC_50_=1.9 μm;**9**, IC_50_=0.75 μm) compared to **M** (IC_50_=10.9 μm) (Table 2). This indicates that rigidifying the diphenylmethane moiety as a tricyclic improves target binding.

**Table 2:** Results of IC_50_ and EC_50_ value determinations of compounds related to the parent molecule **M** (GBR-12935).

Compd^[a]^	TryR IC_50_ [μm]	GR IC_50_ [μm]	*T. brucei* EC_50_ [μm]
**1**	>100	ND^[b]^	>100^[c]^
**2**	>100	ND	51.3±1.6
**3**	82.9±15.5	ND	32.1±1.01
**4**	29.7±1.9	ND	27.9±0.5
**5**	48.7±4.0	ND	13.5±0.7
**6**	51.5±7.9	ND	9.32±1.12
**7**	1.94±0.03	>100	3.50±0.27
**8**	26.9±3.7	>100	2.17±0.12
**9**	0.75±0.06	>100	0.775±0.031
1-(3-phenylpropyl)piperazine	34.5±3.8	ND	ND
1-propylbenzene	>100	ND	ND
3-phenylpropylamine	>100	ND	ND

[a] Compounds synthesised in this paper are shown as their compound number, other compounds names are given in full. [b] ND: not determined. [c] Values shown as >100 μm showed less than 20 % inhibition at a concentration of 100 μm.

**Figure 3:**
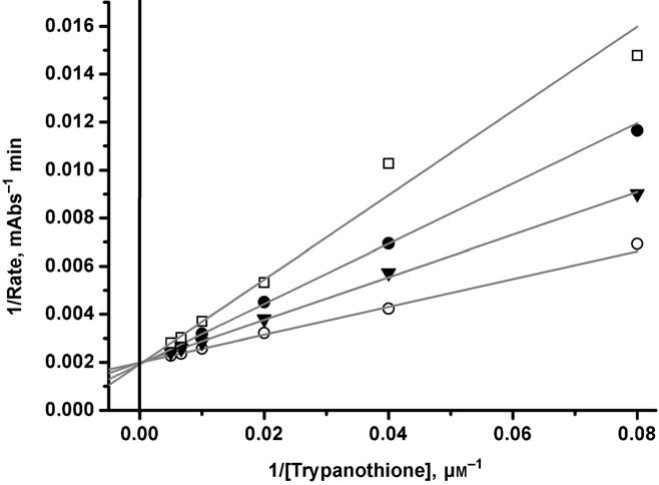
Determination of the inhibition constant (*K*_i_) for compound **9**. A Lineweaver–Burke plot of compound **9** with recombinant TryR from *T. cruzi. K*_i_ was calculated to be 331±19 nm. Points shown are the mean of three measurements at six different substrate concentrations, the lines shown are the least squares fits to the points weighted to one over the standard deviation squared. The concentration of compound **9** was varied (○, 0; ▾, 188; •, 375; □, 750 nm) with the concentration of NADPH being saturating (>10 times *K*_m_).

The presence of the phenylpropyl moiety increased activity significantly against the enzyme (cf. **6** and **7**; **5** and **M**), suggesting that the phenylpropyl moiety has an interaction in the binding site, as has been previously seen in the polyamine class of inhibitors.^19^ The increase in binding potency of the phenylpropyl group can also been seen in the increase in potency when added to the clomipramine (**E**) or chlorpromazine (**H**) scaffolds. In particular the replacement of the *N-*methyl group in prochlorperazine (**K**) by a 3-phenylpropyl moiety caused a tenfold decrease in the IC_50_ value for this compound (cf. **K**, IC_50_=7.46 μm and **9**, IC_50_=0.750 μm), highlighting the important role played by this moiety. Addition of this moiety did not effect the specificity of this inhibitor against human GR (IC_50_>100 μm). The successful synthesis of an improved submicromolar inhibitor of TryR led us to determine the *K*_i_ value and the mode of inhibition of compound **9**, revealing linear competitive inhibition with a *K*_i_ value of 331±19 nm (Figure 3).

In contrast to compound **7**, compound **8** showed a decrease in potency against TryR compared with **M** (**M**, IC_50_=10.9 μm;**8**, IC_50_=26.9 μm, Table 2) suggesting that the phenylpropyl chain is better at binding in this position than the 1-cinnamyl chain.

Compound **M** can be subdivided into two moieties: the diphenylmethane or tricyclic moiety and the phenylpropyl moiety. Both of these individual fragments of compound **M** showed some activity on their own, albeit at a reduced level. Thus 1-(3-phenylpropyl)piperazine gave an IC_50_ value of 34.5 μmagainst TryR (Table 2), which again points to a phenylpropylpiperazine pocket being present in the active site. The specificity of this phenylpropylpiperazine pocket is illustrated by the fact than piperazine itself, propylbenzene, 3-phenylpropylamine and 1-bromo-3-phenylpropane only bind very weakly to the enzyme (IC_50_>100 μm). Similarly, the diphenylmethyl and dibenzosuberyl containing compounds (**3** and **4**) both showed weak activity (IC_50_=82.9 and 29.7 μm, respectively). Again, the stronger binding of compound **4** compared with **3** suggests the greater ability of the more rigid ring system to bind in the tricyclic pocket of TryR.^25^, ^39^

### Cell-based assays on compounds **1–9**

The EC_50_ values of compounds **1**–**9** were determined in the same manner as in the initial screening, and seven of these were found to be lower than the corresponding IC_50_ values against TryR. This suggests, as was found in the initial screenings, that once again these compounds have additional off-target activity or may be selectively concentrated/metabolically activated or a combination of these. Nonetheless, when the IC_50_ values against TryR were plotted against the EC_50_ values against live *T. brucei* parasites, the coefficient of determination (*R*^2^) was 0.72, suggesting a reasonable correlation between enzyme and cell activity. The lowest EC_50_ value was obtained for compound **9** (EC_50_=0.775±0.031 μm), which is the third lowest measured in this study, and the best for a compound considered to be druglike (compound **9**, drug score=0.57).^53^ This EC_50_ value represents a 12-fold improvement from the model compound **M**, which itself is 2.65 times less potent than the ring-joined model compound **7**, proving its relationship to the tricyclic neuroleptic class of inhibitors, and in part confirming the principal of the phenylpropyl moiety improving efficacy.

## Conclusions

The screening of the LOPAC1280 library against TryR and retesting of hits against human GR and *T. brucei* parasites revealed three new classes of TryR inhibitors **J**, **D** and **M** worthy of further development. The screening also revealed that there is no clear relationship between IC_50_ and EC_50_ values for the 22 inhibitors where these were measured, revealing that off-target effects, selective concentration/metabolic activation, or a combination of these factors affect some of these inhibitors.

The inhibitor **M** has been shown to mimic the tricyclic neuroleptic class of inhibitor, where replacement of the diphenylmethane with a 10,11-dihydro-5*H*-dibenzo[*a,d*]cycloheptene ring system caused a fivefold decrease in IC_50_ value of the inhibitor, placing it in the same potency range as the tricyclic class of inhibitors. The information that the active site of the inhibitor may possess a phenylpropyl pocket near where the tricyclic class of inhibitor binds resulted in the synthesis of a prochlorperazine congener with a tenfold decrease in IC_50_ value. In the cases where improved inhibitors have been synthesised (**7** and **9**) lack of activity against human GR (IC_50_>100 μm) has been retained.

## Experimental Section

### Overview of screening

Compounds were initially screened at a concentration of 100 μm in 96-well plates. Positive (1 % *v*/*v* DMSO) and negative (100 μm clomipramine) controls were included on each plate. Plates were assayed in duplicate. Compounds exhibiting >50 % inhibition at 100 μm were re-screened at a concentration of 10 μm in duplicate. The IC_50_ values were determined in triplicate for the top 37 most potent compounds, and the 22 most potent of these were assayed in triplicate against *T. brucei* in vitro to determine the EC_50_ values. In addition, the same 22 compounds were screened against human GR and their IC_50_ values determined whenever possible.

#### Screening against trypanothione reductase

The assay is based on the colorimetric reduction of 5,5′-dithiobis-(2-nitrobenzoic acid) (DTNB) by T[SH]_2_.^37^ The assay mixture consisted of: 40 mm HEPES pH 7.4, 1 mm EDTA, 6.0 μm T[S]_2_, 50 μm DTNB, 2 mU mL^−1^ TryR and 150 μm NADPH. Initial screens at 100 μm were completed in duplicate in 96-well plates with DMSO (1 % *v*/*v*, final concentration) as a positive control (column 1) and clomipramine (100 μm) as a negative control (column 12). An amount of inhibitor (1.8 μL, 10 mm stock in DMSO) was added into each of the wells (100 μm final concentration) and to this was added 158.2 μL of reagents (enzyme+trypanothione+DTNB in assay buffer) and finally 20 μL of NADPH solution to start the assay. Absorbance at 412 nm was monitored at 25 °C for 15 min using a SpectraMax 340PC (Molecular Devices) plate reader. The data was inspected for linearity and the relative percentage inhibitions were calculated from the following equation: Inhibition=(average velocity of inhibitor wells)/(average velocity of 1 % DMSO wells)−(average velocity of clomipramine wells)×100.

IC_50_ values were determined using 11 serial dilutions. Starting with 12 μL of a 10 mm DMSO solution of the inhibitor, 6 μL was removed and added to another Eppendorf tube containing 6 μL of DMSO. The tube was sealed, mixed and then briefly centrifuged to ensure that the sample was at the bottom of the tube. This procedure was repeated 11 times to produce 12 serial 50 % dilutions. Assays were performed in triplicate as described above. Data were inspected for linearity and IC_50_ values determined by nonlinear regression to the following four-parameter equation: *y*=(range/(1+*x*/IC_50_)^Slope factor^)+background. For human GR, essentially the same procedure as above was used with the assay composition as follows: 40 mm HEPES pH 7.4, 1 mm EDTA, 15.0 μm glutathione disulphide, 50 μm DTNB, 5 mU mL^−1^ GR and 150 μm NADPH. The choice of disulphide concentration in both assays represents [S]≈*K*_m_.

#### Cell-based screens

For EC_50_ value determination against bloodstream *T. brucei*, test compounds and the control drug (pentamidine) were dissolved in DMSO at 10 mm and 0.1 mm concentrations, respectively. Drug solutions (2 μL) were added to the second column (B2–G2) of the 96-well plate. Aliquots of 100μL HMI-9 medium^61^ containing 1 % DMSO were added to the rest of the wells and 198 μL HMI-9 medium to the second column (B2–G2). Serial drug dilutions were prepared by withdrawing 100 μL from the second column and adding into the adjacent column. The process was repeated until column 10 was reached. A suspension of trypanosomes was prepared at a density of 2×10^3^ cells mL^−1^ and 100 μL added to each well except column 1, which received 100 μL HMI-9 instead. Columns 1 and 11 served as a control without trypanosomes and a control without drug, respectively. Cells were incubated for 3 days at 37 °C in 5 % CO_2_, after which 20 μL resazurin (0.5 mm) was added to each well and plates incubated for a further 4 h before measuring fluorescence (excitation of 528 nm and emission of 590 nm) on an FLX 800 Fluorescence plate reader (BioTek Instruments). Measurements were obtained on three separate occasions. Data were processed using the GRAFIT program and fitted to the following four-parameter equation: *y*=(range/(1+*x*/EC_50_)^Slope^ ^factor^)+ background, to obtain the effective concentration inhibiting growth by 50 % (EC_50_). The EC_50_ values reported are the weighted means, with the values weighted to the inverse squares of their standard deviations. Pentamidine was used to validate the assay conditions, giving an EC_50_ value of 5.4±0.18 nm, which is lower than the reported value of 14 nm for the S427 strain of *T. brucei*.^54^ The coefficient of variation between assay plates was 3.39 %.

#### Inhibition constant (*K*_i_) determination

The assay solution consisted of: 40 mm HEPES pH 7.4, 1 mm EDTA, 2 mU mL^−1^ TryR and 150 μm NADPH. The rate of NADPH oxidation, monitored at 340 nm at 25 °C using a UV-2401PC UV–vis recording spectrophotometer (Shimadzu Scientific) for six different substrate concentrations (12.5, 25, 50, 100, 150 and 200 μm trypanothione), were measured in triplicate over 30 s for four different inhibitor concentrations (0, 188, 375 and 750 nm). The resulting data was plotted as a Lineweaver–Burke plot to ascertain the mode of inhibition. The data were fitted using weighted nonlinear regression to equations describing both competitive and mixed inhibition. The *K*_i_ value given in Figure 3 is the weighted mean of three determinations of *K*_i_ values for the three inhibitor concentrations used, fitted to equation: *y*=*V*_max_×[Substrate]/*K*_m_×(1+[Inhibitor]/*K*_i_)+[Substrate]), describing competitive inhibition.

### Chemistry

^1^H NMR spectra were recorded on a Brücker spectrometer at 500 MHz in CDCl_3_, ^13^C NMR spectra were recorded in the same solvent on the same spectrometer at 125 MHz, chemical shifts are reported in ppm. Electrospray mass spectra were recorded on a Micromass Q-TOF 2 mass spectrometer (Micromass UK).

**1-(3-Phenylpropyl)piperazine**: A solution of piperazine (10 g, 0.12 mol) and 1-bromo-3-phenylpropane (18.24 mL, 0.12 mol) in EtOH (25 mL) was heated under reflux for 6 h. The solvent was evaporated and the residue was partitioned between H_2_O (125 mL) and Et_2_O (125 mL). The aqueous layer was then extracted with Et_2_O (5×50 mL) and the combined organic fractions were dried (MgSO_4_). The solvent was evaporated to give an oily residue that was purified by distillation (172 °C, 15 mm) to give the title compound as a colourless oil (5.24 g, 23 %): ^1^H NMR (500 MHz, CDCl_3_): *δ*=1.85 (q, *J*=7.1 Hz, 2 H), 2.38 (t, *J*=7.1 Hz, 2 H), 2.45 (m, 4 H), 2.65 (t, *J*=10.3 Hz, 2 H), 2.85 (t, *J*=9.1 Hz, 4 H), 7.16 (t, *J*=7.7 Hz, 1 H), 7.22 (d, *J*=7.6 Hz, 1 H), 7.26 ppm (t, *J*=7.7 Hz, 1 H); ^13^C NMR (125 MHz, CDCl_3_): *δ*=28.0, 33.5, 53.2, 44.9, 58.0, 125.5, 128.0, 128.9, 138.1 ppm; MS (ES, 50 eV): *m*/*z* (%): 204.2 (100), 205.2 (14) [*M*+H]^+^; HRMS-ES: *m*/*z* [*M*+H]^+^ calcd for C_13_H_20_N_2_: 205.16265, found: 205.17254.

**General procedure for the synthesis of 2-substituted chloroethanes 3 and 4**: A mixture of **1** or **2** (54 mmol) and 2-chloroethanol (3.85 mL, 57 mmol) in toluene (28.8 mL) and *p*TsOH (0.344 g, 2 mmol) was refluxed for 1 h using a Dean–Stark trap. The cold solution was washed with H_2_O (15 mL), saturated NaHCO_3_ solution (10 mL), H_2_O (15 mL), dried (MgSO_4_), filtered and evaporated to yield the target compounds as white solids. Compounds were used without further purification.

**(2-Chloroethoxy)diphenylmethane (3)**: (11.66 g, 82 %): ^1^H NMR (500 MHz, CDCl_3_): *δ*=3.74 (t, *J*=6.2 Hz, 2 H), 3.80 (t, *J*=6.2 Hz, 2 H), 5.5 (s, 1 H), 7.33 (t, *J*=8.1 Hz, 1 H), 7.41 (t, *J*=8.1 Hz, 1 H), 7.45 ppm (d, *J*=8.1 Hz, 1 H); ^13^C NMR (125 MHz, CDCl_3_): *δ*=43.2, 69.2, 84.1, 127.1, 128.0, 129.3, 140.3 ppm; MS (ES, 50 eV): *m*/*z* (%): 269.1 (100), 271.1 (32) [*M*+Na]^+^; HRMS-ES: *m*/*z* [*M*+Na]^+^ calcd for C_15_H_15_ClO: 269.07091, found: 269.07164.

**5-(2-Chloroethoxy)-10,11-dihydro-5*H*-dibenzo[*a,d*]cycloheptene (4)**: (11.04 g, 70 %): ^1^H NMR (500 MHz, CDCl_3_): *δ*=3.08 (t, *J*=12.9 Hz, 2 H), 3.65 (t, *J*=12.9 Hz, 2 H), 3.73 (t, *J*=6.6 Hz, 2 H), 3.79 (t, *J*=6.6 Hz, 2 H), 5.50 (s, 1 H), 7.25 (t, *J*=7.8 Hz, 1 H), 7.30 (t, *J*=7.6 Hz, 1 H), 7.45 ppm (d, *J*=7.6 Hz, 1 H); ^13^C NMR (125 MHz, CDCl_3_): *δ*=32.5, 43.5, 68.0, 69.0, 126.9, 128.2, 128.5, 131.0, 132.9, 136.9 ppm; MS (ES, 50 eV): *m*/*z* (%): 295.1 (100), 297.1 (32) [*M*+Na]^+^; HRMS-ES: *m*/*z* [*M*+Na]^+^ calcd for C_17_H_17_ClO: 295.08656, found: 295.08953.

**General procedure for synthesis of the N-substituted piperazines (5 and 6)**: A solution of **3** or **4** (5.42 mmol) in toluene (1.13 mL) was added to a stirred solution of anhyd piperazine (1.396 g, 16.2 mmol) and K_2_CO_3_ (0.56 g, 4.05 mmol) in toluene (15.87 mL). The mixture was refluxed for 15 h, cooled to RT and treated with H_2_O (25 mL). The organic phase was washed twice with H_2_O (2×25 mL) and then extracted with 10 % AcOH (2×20 mL). The combined aqueous acidic solutions were washed with toluene (3×25 mL). The base was liberated with aqueous NaOH and extracted with toluene (3×25 mL). The combined organic fractions were washed with H_2_O, dried (MgSO_4_), filtered and the solvent was evaporated to yield the target compounds as white solids

**1-((2-Benzhydryloxy)ethyl)piperazine (5)**: (853 mg, 53 %): ^1^H NMR (500 MHz, CDCl_3_): *δ*=1.7 (NH), 2.5 (m, 4 H), 2.7 (t, *J*=10.1 Hz, 2 H), 2.9 (t, *J*=7.3 Hz, 4 H), 3.65 (t, *J*=7.3 Hz, 2 H), 7.26 (t, *J*=8.1 Hz, 1 H), 7.33 (d, *J*=8.1 Hz, 1 H), 7.36 ppm (t, *J*=8.1 Hz, 1 H); ^13^C NMR (125 MHz, CDCl_3_): *δ*=46.2, 55.2, 59.0, 67.0, 84.1, 127.2, 127.8, 128.9 ppm; MS (ES, 50 eV): *m*/*z* (%): 296.2 (100), 297.2 (21) [*M*+H]^+^; HRMS-ES: *m*/*z* [*M*+H]^+^ calcd for C_19_H_24_N_2_O: 297.18886, found: 297.18496.

**1-[2-(10,11-Dihydro-5*H*-dibenzo[*a,d*]cyclohepten-5-yloxy)ethyl]piperazine (6)**: (578 mg, 33 %): ^1^H NMR (500 MHz, CDCl_3_): *δ*=2.45 (m, 4 H), 2.65 (t, *J*=10.1 Hz, 2 H), 3.03 (q, *J*=12.9 Hz, 2 H), 3.55 (q, *J*=12.9 Hz, 2 H), 3.60 (t, *J*=7.3 Hz, 2 H) 5.40 (s, 1 H), 7.15 (t, *J*=7.6 Hz, 1 H), 7.20 (t, *J*=7.6 Hz, 1 H), 7.38 ppm (d, *J*=7.6 Hz, 1 H); ^13^C NMR (125 MHz, CDCl_3_): *δ*=32.1, 46.1, 55.0, 58.8, 66.3, 68.5, 125.6, 128.1, 129.1, 130.5, 132.9, 136.5 ppm; MS (ES, 50 eV): *m*/*z* (%): 322.2 (100), 323.2 (23) [*M*+H]^+^; HRMS-ES: *m*/*z* [*M*+H]^+^ calcd for C_21_H_26_N_2_O: 323.20451, found: 323.20477.

**General procedure for synthesis of the N,N-disubstituted piperazines (7 and 8)**: A solution of **3** or **4** (1 g, 3.68 mmol), substituted piperazine (3.7 mmol) and K_2_CO_3_ (0.51 g, 3.7 mmol) in toluene (6 mL) was heated under reflux for 16 h, cooled to RT, washed with H_2_O (2×25 mL) and extracted with toluene (3×25 mL). After column chromatography (silical gel, MeOH) the products were isolated as white solids.

**(1-[2-(10,11-Dihydro-5*H*-dibenzo[*a,d*]cyclohepten-5-yloxy)ethyl]-4-(3-phenyl-propyl)piperazine (7)**: (185 mg, 11 %): ^1^H NMR (500 MHz, CDCl_3_): *δ*=1.85 (q, *J*=7.3 Hz, 2 H), 2.4 (t, *J*=7.3 Hz, 2 H), 2.5 (m, 8 H), 2.63 (t, *J*=7.3 Hz, 2 H), 2.68 (t, *J*=7.2 Hz, 2 H), 3.01 (q, *J*=12.8 Hz, 2 H), 3.55 (q, *J*=12.8 Hz, 2 H), 3.6 (t, *J*=7.2 Hz, 2 H) 5.4 (s, 1 H) 7.1–7.4 ppm (m, 13 H); ^13^C NMR (125 MHz, CDCl_3_): *δ*=28.3, 32.3, 33.8, 53.2, 53.7, 57.8, 58.0, 66.7, 125.9, 126.0, 126.8, 127.0, 127.1, 130.1, 130.2, 132.9, 135.2, 136.5 ppm; MS (ES, 50 eV): *m*/*z* (%): 463.3 (100), 464.3 (33) [*M*+Na]^+^; HRMS-ES: *m*/*z* [*M*+Na]^+^ calcd for C_30_H_36_N_2_O: 463.27253, found: 463.27123.

**1-[2-(10,11-Dihydro-5*H*-dibenzo[*a,d*]cyclohepten-5-yloxy)ethyl]-4-(*E*)-3-phenyl-allyl)piperazine (8)**: (297 mg, 18 %): ^1^H NMR (500 MHz, CDCl_3_): *δ*=2.53 (m, 8 H), 2.65 (t, *J*=7.3 Hz, 2 H), 3.0 (q, *J*=7.3 Hz, 2 H), 3.2 (d, *J*=7.8 Hz, 2 H), 3.55 (q, *J*=12.7 Hz, 2 H), 3.6 (t, *J*=12.7 Hz, 2 H), 5.4 (s, 1 H), 6.3 (t, *J*=15.9 Hz, 1 H), 6.55 (d, *J*=15.9 Hz, 1 H), 7.1–7.4 ppm (m, 13 H); ^13^C NMR (125 MHz, CDCl_3_): *δ*=32.2, 53.1, 53.4, 58.0, 60.8, 66.6, 68.5, 125.9, 126.4, 126.6, 127.5, 128.0, 128.6, 128.7, 130.3, 132.9, 133.1, 135.2, 136.5 ppm; MS (ES, 50 eV): *m*/*z* (%): 461.3 (100), 462.3 (32) [*M*+Na]^+^; HRMS-ES: *m*/*z* [*M*+Na]^+^ calcd for C_30_H_34_N_2_O: 461.25688, found: 461.27478.

**2-Chloro-10-(3-(4-(3-phenylpropyl)piperazin-1-yl)propyl)-10*H*-phenothiazine (9)**: A mixture of 2-chlorophenothiazine (1.17 g, 5 mmol) and powdered KOH (0.29 g, 5 mmol) in dry DMF (10 mL) was stirred under N_2_ for 30 min. The alkyl bromide 1,3-dibromopropane (3.03 g, 15 mmol) was introduced and the mixture was stirred for 48 h at RT. The mixture was poured into H_2_O (50 mL) and extracted with CH_2_Cl_2_, washed with H_2_O (2×50 mL), separated and dried (MgSO_4_). After removal of the solvent, the crude product was redissolved in toluene (10 mL) and treated with 1-(3-phenylpropyl)piperazine (1.02 g, 5 mmol) and K_2_CO_3_ (0.69 g, 5 mmol). The reaction was heated under reflux for 48 h, cooled to RT, washed with H_2_O (2×25 mL) and extracted with toluene (3×25 mL). After column chromatography the product was isolated as a white solid (120.4 mg; 5 %): ^1^H NMR (500 MHz, [D_6_]DMSO): *δ*=1.69 (p, *J*=7.3 Hz, 2 H), 1.97 (p, *J*=7.5 Hz, 2 H), 2.26 (t, *J*=7.3 Hz, 4 H), 2.51 (d, *J*=8.1 Hz, 8 H), 2.79 (t, *J*=7.3 Hz, 2 H), 3.72 (t, *J*=7.5 Hz, 2 H), 6.73 (d, *J*=7.4 Hz, 1 H), 6.75 (d, *J*=7.4 Hz, 1 H), 6.77 (t, *J*=7.4 Hz, 1 H), 6.79 (t, *J*=7.4 Hz, 1 H), 6.90 (d, *J*=7.4 Hz, 1 H), 6.91 (d, *J*=7.6 Hz, 1 H), 6.99 (t, *J*=7.6 Hz, 1 H), 7.15 (t, *J*=7.6 Hz, 1 H), 7.18 (d, *J*=7.6 Hz, 2 H), 7.25 ppm (t, *J*=7.6 Hz, 2 H); ^13^C NMR (125 MHz, [D_6_]DMSO): *δ*=28.4, 32.0, 33.3, 37.4, 43.0, 44.9, 52.7, 57.9, 114.1, 114.2, 115.1, 115.2, 121.6, 122.8, 126.1, 126.7, 127.9, 128.2, 128.7, 128.7, 145.1, 146.8 ppm; MS (ES, 50 eV): *m*/*z* (%): 500.2 (100), 502.2 (32) [*M*+Na]^+^; HRMS-ES: *m*/*z* [*M*+Na]^+^ calcd for C_28_H_32_ClN_3_S: 500.19032, found: 500.18676.
